# Annotated draft genome sequences of *Mucor flavus* KT1a and *Helicostylum pulchrum* KT1b strains isolated from dry-aged beef surface

**DOI:** 10.1128/mra.00343-24

**Published:** 2024-07-31

**Authors:** Misa Hosono, Midori Torimaru, Kenji Fukuda, Nana Mikami, Takahito Toyotome

**Affiliations:** 1 Department of Life and Food Sciences, Obihiro University of Agriculture and Veterinary Medicine, Obihiro, Hokkaido, Japan; 2 Research Center for Global Agromedicine, Obihiro University of Agriculture and Veterinary Medicine, Obihiro, Hokkaido, Japan; 3 Department of Veterinary Medicine, Obihiro University of Agriculture and Veterinary Medicine, Obihiro, Hokkaido, Japan; 4 Diagnostic Center for Animal Health and Food Safety, Obihiro University of Agriculture and Veterinary Medicine, Obihiro, Hokkaido, Japan; 5 Medical Mycology Research Center, Chiba University, Chiba, Japan; University of Maryland School of Medicine, Baltimore, Maryland, USA

**Keywords:** dry-aged beef, *Mucor flavus*, *Helicostylum pulchrum*

## Abstract

*Mucor flavus* KT1a and *Helicostylum pulchrum* KT1b were isolated and identified in our earlier study as the two dominant fungal species on dry-aged beef. In this study, we report their genome sequences and annotations.

## ANNOUNCEMENT

Dry-aged beef (DAB) is a processed meat product that offers unique flavors and enhances the taste. *Mucor flavus* KT1a and *Helicostylum pulchrum* KT1b have been identified as the dominant fungal species on DAB surface (1). In this study, we report their genome sequences.

The strains were cultured in potato dextrose broth at 15°C for 7 days with shaking at 100 rpm. Hyphae were recovered, frozen in liquid N_2_, and crushed. Fragments were suspended in lysis buffer (2 mL) supplemented with RNase A (20 µg/mL). After 30 min, they were treated with Proteinase K (0.8 mg/mL) at 50°C for 2 h. DNA purification was performed using a Genomic-Tip 20/G (Qiagen, Hilden, Germany). The resultant DNA was resuspended in Tris-EDTA buffer (pH 8.0). Buffer SRE XS Kit (Circulomics, Inc., Baltimore, MD, USA) was used to eliminate low-molecular-weight DNA. The samples were sequenced by the Bioengineering Lab. Co., Ltd. (Kanagawa, Japan). DNA samples were repurified using 1.8× solution vol of AMPure XP reagent (Beckman Coulter, Inc., Brea, CA, USA) and fragmented to approximately 15 kbp using g-TUBE (Covaris, LLC., Woburn, MA, USA). Libraries from ultra-low DNA inputs were obtained using the SMRTbell gDNA Sample Amplification Kit and SMRTbell Express Template Prep Kit 2.0 (PacBio, Menlo Park, CA, USA), following the instructions outlined in documentations provided by PacBio. Subsequently, the libraries were sequenced using the Binding Kit 2.2 with Sequel IIe (PacBio). To align the subreads, overhang adaptor sequences were eliminated using the SMRT Link (v.12.0.0.177059 and 11.0.0.146107 for KT1a and KT1b, respectively). Consensus sequences were obtained from the sub-read data, and those exhibiting quality value ≥20 were used as high-fidelity (HiFi) reads for subsequent analysis. Ultra-low PCR adaptors were eliminated using lima (ver. 2.7.1 and 2.6.0 for KT1a and KT1b, respectively) (2), followed by the elimination of duplicate PCR reads using pbmarkdup (ver. 1.0.3 and 1.0.2 for KT1a and KT1b, respectively) (3) from the HiFi reads. Reads with <1,000 bp were eliminated using Filtlong (ver. 0.2.1) (4). The resultant reads were assembled using the IPA software (ver. 1.8.0) (5), and the completeness of the genome assembly was assessed using the “eukaryote” model by BUSCO (ver. 5.4.6_cv1 and 5.4.4_cv1 for KT1a and KT1b, respectively) (6). The assembled sequences were soft masked using RepeatMasker (Galaxy Version 4.1.5+ galaxy0) (7). Gene prediction and annotation were performed using Funannotate software (Galaxy Version 1.8.15 + galaxy1) (8). The following parameters, --busco_db mucolares (orthodb 10) and --busco_seed_species Rhizopus_oryzae, were changed from the default settings.

Sequencing of KT1a revealed 171,722 reads with an average length of 7,270 bp and 148,292 HiFi reads. For KT1b, 217,295 reads were obtained, with an average length of 5,319 bp and 188,727 quality-filtered reads. The assembled genomes are presented in Table 1. The number of predicted genes in KT1a and KT1b is 11,530 and 12,200, respectively.

**TABLE 1:** The statistics and the summary of analysis by BUSCO of assembled genomes of *M. flavus* KT1a and *H*. *pulchrum* KT1b

Species strain	Read N_50_ (bp)	Contig	Contig N_50_ (bp)	The longest contig	Total (bp)	GC contents (%)	Coverage	BUSCO			
								Complete and single copy	Complete and duplicated	Fragmented	Missing
*M. flavus* KT1a	7,270	69	857,634	3,272,716	33,728,218	34.6	37.0	240	6	7	2
*H. pulchrum* KT1b	5,319	91	946,389	2,241,229	34,742,669	34.2	33.3	236	10	6	3

## Data Availability

The sequencing data obtained were deposited in the DNA Data Bank of Japan/European Nucleotide Archive/Genetic Sequence Database (DDBJ/ENA/GenBank) under accession no. DRR531250 and DRR531249 for KT1a and KT1b, respectively. Annotation data have been deposited under accession no. BAABUK010000001-BAABUK010000069 and BAABUJ010000001-BAABUJ010000091 for KT1a and KT1b, respectively.
